# Early Austronesians Cultivated Rice and Millet Together: Tracing Taiwan’s First Neolithic Crops

**DOI:** 10.3389/fpls.2022.962073

**Published:** 2022-07-22

**Authors:** Zhenhua Deng, Su-chiu Kuo, Mike T. Carson, Hsiao-chun Hung

**Affiliations:** ^1^Center for the Study of Chinese Archaeology, Peking University, Beijing, China; ^2^School of Archaeology and Museology, Peking University, Beijing, China; ^3^Institute of History and Philology, Academia Sinica, Taipei, Taiwan; ^4^University of Guam, Mangilao, GU, United States; ^5^Department of Archaeology and Natural History, The Australian National University, Canberra, ACT, Australia

**Keywords:** Austronesian, mixed farming, *japonica* rice, the Zhiwuyuan (Botanical Garden) site, northern Taiwan

## Abstract

This study presents the first directly dated physical evidence of crop remains from the Early Neolithic archaeological layers in Taiwan. Systematic sampling and analysis of macro-plant remains suggested that Neolithic farmers at the Zhiwuyuan (Botanical Garden) site in Taipei, northern Taiwan, had cultivated rice and foxtail millet together at least 4,500 years ago. A more comprehensive review of all related radiocarbon dates suggests that agriculture emerged in Taiwan around 4,800–4,600 cal. BP, instead of the previous claim of 5,000 cal. BP. According to the rice grain metrics from three study sites of Zhiwuyuan, Dalongdong, and Anhe, the rice cultivated in northern and western-central Taiwan was mainly a short-grained type of the *japonica* subspecies, similar to the discoveries from the southeast coast of mainland China and the middle Yangtze valley. These new findings support the hypothesis that the southeast coast of mainland China was the origin of proto-Austronesian people who brought their crops and other cultural traditions across the Taiwan Strait 4,800 years ago and eventually farther into Island Southeast Asia.

## Introduction

The early dispersal of Austronesian-speaking populations marked one of the world’s most extensive human migrations, giving rise to the most widely distributed language family before the colonial period (Blust, 1984; Kikusawa, 2014). In the past decades, several hypotheses have been proposed to interpret the rhythm and mechanism of this extraordinary event (e.g., Solheim, 1975; Bellwood, 1984; Diamond, 1988; Meacham, 1988; Oppenheimer and Richards, 2001). With the progress of archaeological, linguistic, and genetic studies, recent research has generated increasing evidence supporting the *Out of Taiwan* hypothesis (the homeland) and the *Farming-Language Dispersal* model (the migration mechanism) for Austronesian dispersal, first from Taiwan into the northern Philippines around 4,200 years BP, and onward into the Mariana Islands, Indonesian Archipelago, the Bismarck Archipelago, and beyond by 3,500 BP (e.g., Bellwood, 2002, 2005; Hung, 2005, 2008; Gray et al., 2009; Summerhayes, 2010; Hung et al., 2011; Carson et al., 2013; Skoglund et al., 2016; McColl et al., 2018; Blust, 2019; Chambers and Edinur, 2021; Pugach et al., 2021). One of the critical issues of these debates is whether agriculture dispersed contemporary with the early Austronesian migrants (Latinis, 2000; Paz, 2005; Denham, 2013). However, the main reason for these disputes is that very few archaeobotanical works have been completed in the region.

Rice has been in the central place of debates on the Austronesian expansion; whether the dispersal of rice followed the same pace as the early migrants from the southeast coast of mainland China into Taiwan and then Island Southeast Asia has been a widely disputed subject, especially for debates of the *Out of Taiwan* hypothesis and *Farming-Language Dispersal* model (Donohue and Denham, 2010; Bellwood, 2011). Moreover, some recent attempts based on genome analysis of modern rice landraces suggested a very different dispersal route and process (Gutaker et al., 2020; Alam et al., 2021), making this situation even more puzzling.

By contrast, new archaeobotanical data and radiocarbon dating tend to support a simultaneous dispersal of Austronesian people with their Neolithic package of plant and animal domestications, pottery and ground stone manufacturing, weaving, and other Neolithic innovations (e.g., Bellwood, 2011, 2017; Anggraeni et al., 2014; Chang et al., 2015; Deng et al., 2020a; Hung et al., 2022).

From the Early to Middle Neolithic archaeological contexts (4,800 through 3,500 cal. BP), Taiwan witnessed a rapid development of local society. The total number of settlement sites in the period of 4,500–3,500 cal. BP was more than seven times that of the previous period (Tsang, 1990; Hung and Carson, 2014). Concurrently, cultural groups developed regional diversity, as seen in the newly emerging cultural assemblages in different areas: Xuntangpu in northern Taiwan, Niumatou in central-western Taiwan, Niuchouzi in southern Taiwan, and Fushan in eastern Taiwan (Liu, 2007a; Hung and Carson, 2014; Kuo, 2019). During this period, some people moved to the Batanes and northern Luzon from Taiwan, starting the protracted dispersal in the following thousands of years (Hung, 2005, 2008; Bellwood et al., 2011; Bellwood and Dizon, 2013; Carson and Hung, 2018). Consequently, the Early-Middle Neolithic subsistence strategy in Taiwan is pivotal for understanding the economic foundation of early Formosan society and resolving current debates on Austronesian dispersal.

So far, somewhat limited archaeobotanical analyses have been conducted in Taiwan. Before this study, the Nanguanlidong site in southwestern Taiwan yielded the most abundant crop remains and suggested that rice (*Oryza sativa*), foxtail millet (*Setaria italica*), and broomcorn millet (*Panicum miliaceum L.*) were cultivated together (Tsang, 2005; Hsieh et al., 2011; Tsang et al., 2017). Additionally, different morphotypes of rice phytoliths around 4,200 cal. BP have been recovered from the Chaolaiqiao site in eastern Taiwan (Deng et al., 2018a). Otherwise, only a few rice grains have been reported from limited sites of Taiwan. More importantly, until now, no direct dating has been acquired from the actual ancient crops in any part of the island (including Nanguanlidong), making the exact timing ambiguous for the first farming in Taiwan.

The rich, intact rice grains from Zhiwuyuan, Dalongdong, and Anhe have provided an opportunity to investigate these questions. The critical time span of the earliest layers of these sites was contemporary to Nanguanlidong, although different terminologies were applied to name their affiliated cultural assemblages (e.g., Tsang, 2005; Hung and Carson, 2014; Kuo, 2019). However, like Nanguanlidong, all bottom layers of the three sites contained the diagnostic pottery of the Dabenkeng tradition (also known as Tapenkeng or TPK, see Chang, 1969), an essential cultural index of the early Neolithic in Taiwan. The present study involved a systematic analysis of macroscopic plant remains, as well as direct dating of those ancient plant remains, at the three key sites in northern and central-western Taiwan. New evidence from this study now can contribute toward clarifying the debates and questions about the emergence of agriculture in Taiwan.

## Materials and Methods

### Site Description of Three Study Sites

**Figure 1:**
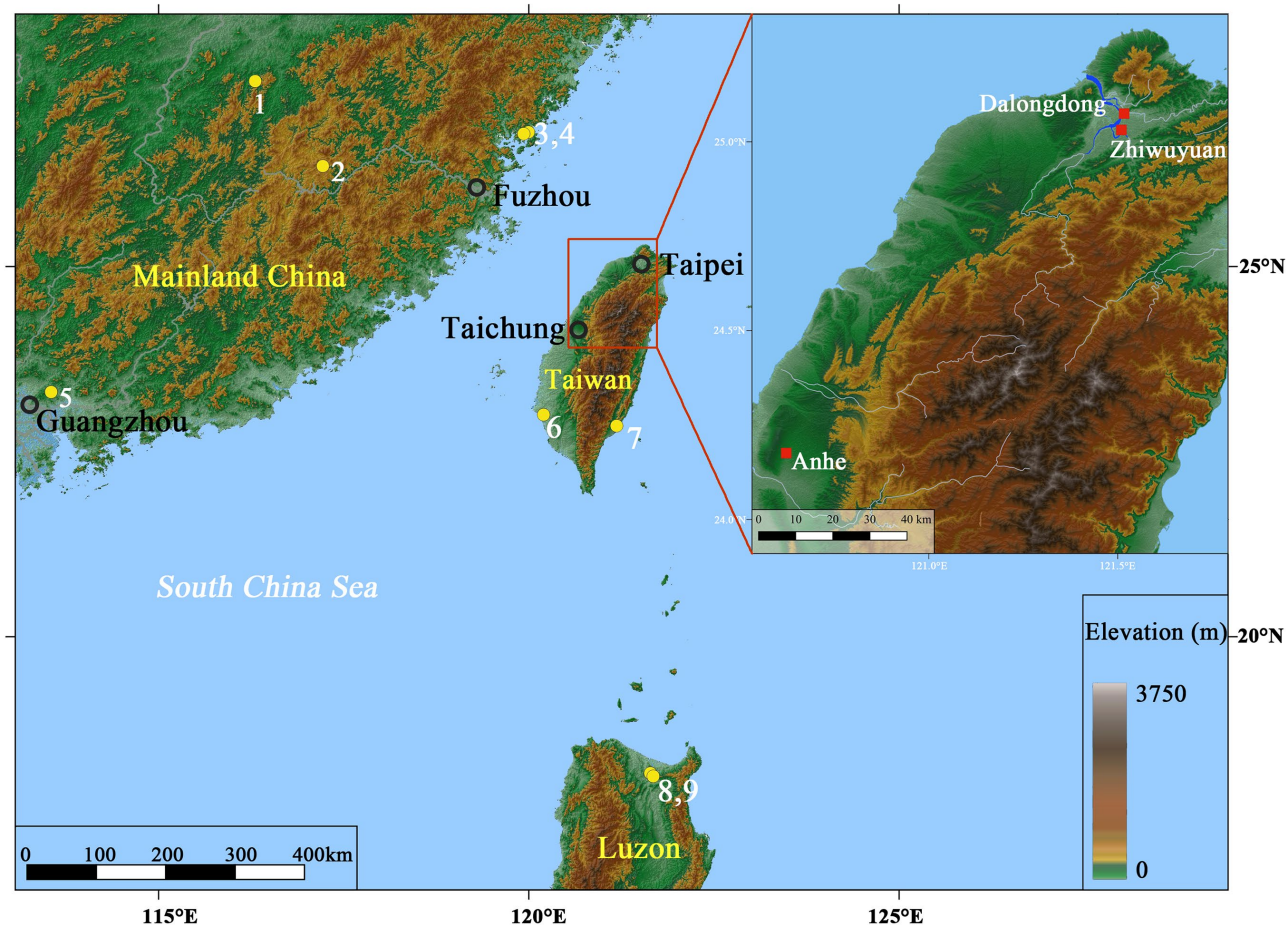
Location of the studied sites and other main sites mentioned in this study; (1) Guodishan, (2) Nanshan, (3) Huangguashan, (4) Pingfengshan, (5) Gancaoling, (6) Nanguanlidong and Nanguanli, (7) Chaolaiqiao, (8) Nagsabaran, and (9) Magapit.

The Zhiwuyuan site (120°30′37′E, 25°01′54′N) is situated in the central area of Taipei city (Figure 1). The north part of the site is within the scope of Taipei Botanical Garden (*Zhiwuyuan*), and other modern buildings and roads cover the south part. This region is the north terrace of the Xindian River, a tributary of the Danshui River in the Taipei Basin. The elevation of the site is around 6–8 m above sea level in modern times. It was discovered in 1901 and primarily surveyed in 1943 (Sato, 1901; Kanaseki and Kokubu, 1954; Kokubu, 1981), and then, several systematic surveys and excavations were conducted from 1999 to 2018 (Liu and Kuo, 2000; Chen and Kuo, 2004; Liu et al., 2006; Huang et al., 2008; Chiu et al., 2010; Liu, 2011; Kuo, 2021).

These efforts revealed that the whole site covers an area of roughly 60,000 m^2^, and the depth of the cultural deposit (in most parts of the site) is almost 5 m. The cultural remains of Zhiwuyuan are divided into four periods (or “phases”) by the excavator Kuo (2021), from the “Early Xuntangpu cultural phase” (*ca.* 5,200/4,800–4,200 cal. BP), and then, the next periods were described as phases of Yuanshan (3,000–2,500 cal. BP), Zhiwuyuan (2,300–1,800 cal. BP), and Shisanhang (1,400–1,100 cal. BP), with several hiatus periods between them. The “Early Xuntangpu cultural phase,” as defined by Kuo (2021), may have overlapped with the traditionally defined “Dabenkeng (or Tapenkeng) cultural phase” (e.g., Chang, 1969; Tsang, 2005; Hung and Carson, 2014), due to the range of the radiocarbon dating and typical Dabenkeng pottery style from this cultural layer (see the section Discussion below).

The Dalongdong site (121°31′00′E, 25°04′26′N) is located in Taipei city, only 5 km to the north of Zhiwuyuan (Figure 1). The site was discovered in 2006 during the demolition of modern buildings in this area (Liu, 2007b). The whole area of the site is over 25,000 m^2^. A test excavation (less than 100 m^2^) was conducted at Dalongdong in 2007, and a large-scale rescue excavation opened nearly 7,600 m^2^ in 2009 (Chu, 2012). As a result, many pits with refilled daily refuse and 20 artificial ditches were discovered. This excavation yielded abundant and varied pottery types and stone tools, as well as a few plant remains such as rice grains and fruit stones of chinaberry (*Melia azedarach*). Eight charcoal samples from Dalongdong have been dated by radiocarbon (Liu, 2007b; Chu, 2012), wherein the calibrated results concentrated in 4,500–4,100 cal. BP, with the early boundary extending at a low probability perhaps as old as 4,800 cal. BP.

The Anhe site (24°10′32′N, 120°37′21′E) is so far the earliest known Neolithic site in central Taiwan (Figure 1). It was discovered in 2003 and then excavated in 2014 and 2015, including reported excavations of 1,400 m^2^ and 12 m^2^, respectively (Chu, 2016; Yang and Cai, 2016). A cemetery of 48 human burials was found within 400 m^2^ in the south part of the 2014 excavation area. Another 10 pits in the same area contained unique artifacts, such as double conjoined cups, jars, and jade ornaments, which are believed to be associated with sacrificial practices. More than 4,000 ceramics and large amounts of stone tools, jade artifacts, and animal bones have been recovered from this site. A few plant remains were unearthed in 2015, including 11 charred rice grains and fragments. Eight radiocarbon dates from the 2014 excavation (Chu, 2016) concentrated around 4,800–4,000 cal. BP, and only one date was much older than the others at 5,654–5,479 cal. BP (95.4% probability).

### Sample Collection and Processing

**Figure 2:**
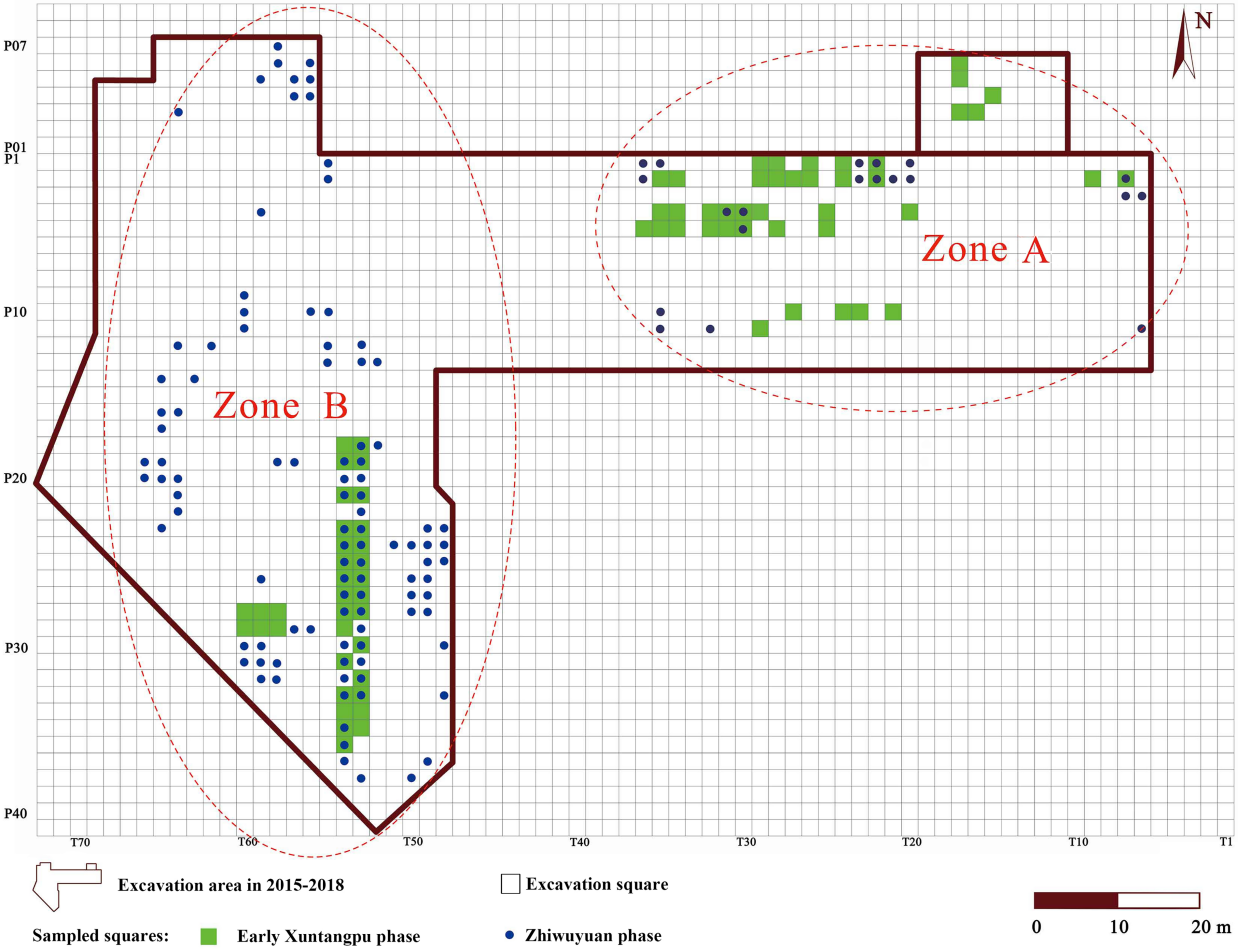
Layout of the excavation area and the distribution of sampled squares of the Zhiwuyuan site in 2015–2018.

Soil samples were collected during the excavation at Zhiwuyuan from 2015 to 2018. Because the excavation area is too large (6,461 m^2^), only a portion of excavation squares was selected for sampling (see Figure 2). The sampling area of Zhiwuyuan could be divided into two separate spatial zones: Zone A in the west and Zone B in the east. All samples were retrieved from measured excavation squares (2 × 2 m^2^) and depth levels (every 10 cm).

Based on analysis of artifacts and radiocarbon dates, Neolithic cultural deposits of the 2015–2018 excavation area could be divided into the Early Xuntangpu phase and the Zhiwuyuan phase. In total, 101 samples of the Early Xuntangpu phase and 217 samples of the Zhiwuyuan phase have been collected (Supplementary Table S1). These samples were floated at the site using buckets, and macroscopic plant remains were retrieved by mesh bags with 300 × 300 μm^2^ apertures. All samples were dried in the shade at the site and sent to the Archaeobotanical Laboratory of Peking University. Seeds, fruits, and other plant remains were sorted, identified, and counted under the microscope at 15–20 magnification, referring to modern references and published criteria (Wang, 1990; Nesbitt, 2005; Guo, 2009; Cappers and Bekker, 2013). In addition, the length, width, and thickness of intact mature rice grains were measured under a microscope.

Rice grains from the 2009 excavation at Dalongdong and the 2015 excavation at Anhe were re-examined with the permission of the Institute of History and Philology, Academia Sinica. All intact specimens were measured under the microscope for grain metrics analysis in Academia Sinica. In this regard, 19 rice grains from Dalongdong and four rice grains from Anhe were documented carefully.

Nine samples from the Early Xuntangpu and one from the Zhiwuyuan phase of the Zhiwuyuan site have been processed at the Key Laboratory of Radiocarbon Dating in Peking University and Beta Analytical for accelerator mass spectrometry (AMS) radiocarbon dating. These samples include two foxtail millet grains, one rice spikelet base, and seven rice grain fragments. In addition, three rice grains from Dalongdong were directly dated at the Radiocarbon Laboratory in the Australian National University. Details of all dated samples are presented in Supplementary Table S2.

## Results

### Radiocarbon Dating Results

**Figure 3:**
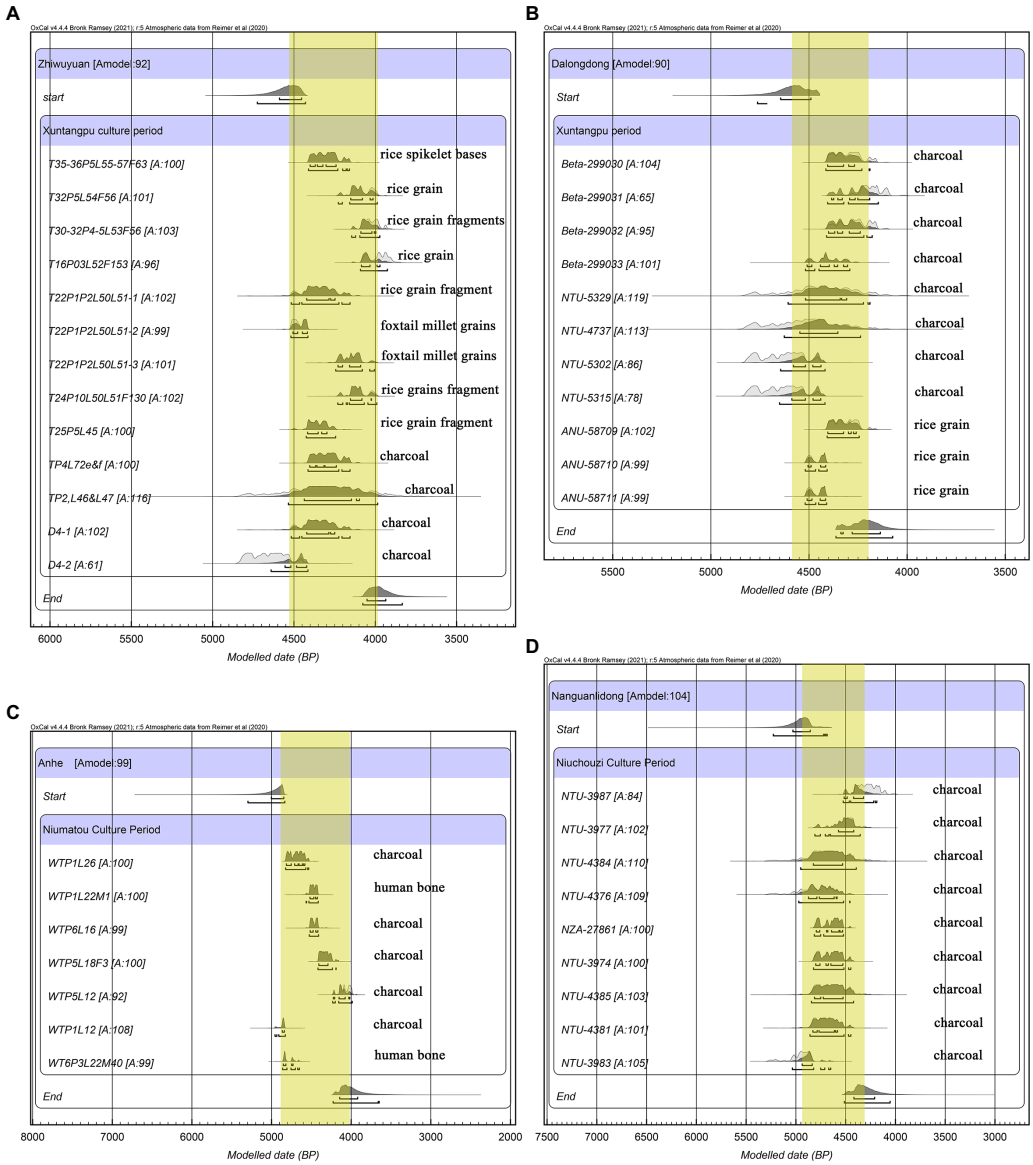
Radiocarbon dates from Zhiwuyuan **(A)**, Dalongdong **(B)**, Anhe **(C)**, and Nanguanlidong **(D)**, calibrated in a Bayesian model incorporating phasing by OxCal 4.4 (Bronk Ramsey, 2009) using the IntCal20 atmospheric curve (Reimer et al., 2020; References of previous data and details of all data are presented in Supplementary Table S2).

In total, this study obtained 12 direct radiocarbon dates of the crop remains from Zhiwuyuan and Dalongdong (Supplementary Table S2). All dates were calibrated along with previously published dates of the same site in a Bayesian model incorporating phasing by OxCal 4.4 (Bronk Ramsey, 2009), using the IntCal20 atmospheric curve (Reimer et al., 2020). The eight dates of the Early Xuntangpu culture phase from the Zhiwuyuan site are generally consistent with their cultural affiliations, concentrated in 4,520–4,000 cal. BP. Before this study, four dates of this period were published (Chen and Kuo, 2004; Liu et al., 2006; Chiu et al., 2010), but three were based on conventional radiocarbon dating with a wide error range, the older limit of which thus could extend as old as 4,830 cal. BP. However, according to the Bayesian model, the Early Xuntangpu culture period of Zhiwuyuan most likely began around 4,534 cal. BP and ended around 3,986 cal. BP (using medium age of boundary start and end date, see Figure 3A). The rice grain sample from the Zhiwuyuan cultural phase yielded a date of 1,786–1,618 cal. BP, slightly later than previously suggested dating (2,300–1,800 cal. BP; Kuo, 2021, for original dates, see Supplementary Table S2).

Two rice grains from Dalongdong yielded similar dates, including one at 4,520–4,409 cal. BP and another at 4,520–4,411 cal. BP (95.4% probability). A third date was slightly later, at 4,410–4,189 cal. BP (95.4% probability). These three direct dates from rice remains are consistent with the previous four AMS radiocarbon dates from charcoal samples (Liu, 2007b; Chu, 2012). However, the four conventional radiocarbon dates of charcoal yielded a wider range, with the older limit reaching 4,820 cal. BP. Bayesian model analysis of all these dates indicates that the occupation period of Dalongdong began around 4,585 cal. BP and ended around 4,208 cal. BP (using medium age of boundary start and end date, see Figure 3B).

Regarding the Anhe site, no direct dates of crops have been obtained in this study. However, our Bayesian analysis of previously published dates reveals that Anhe possibly was occupied slightly earlier than the other two sites, extending most likely from 4,943 to 4,010 cal. BP (using medium age of boundary start and end date, see Figure 3C).

### Macroscopic Plant Remains

**Figure 4:**
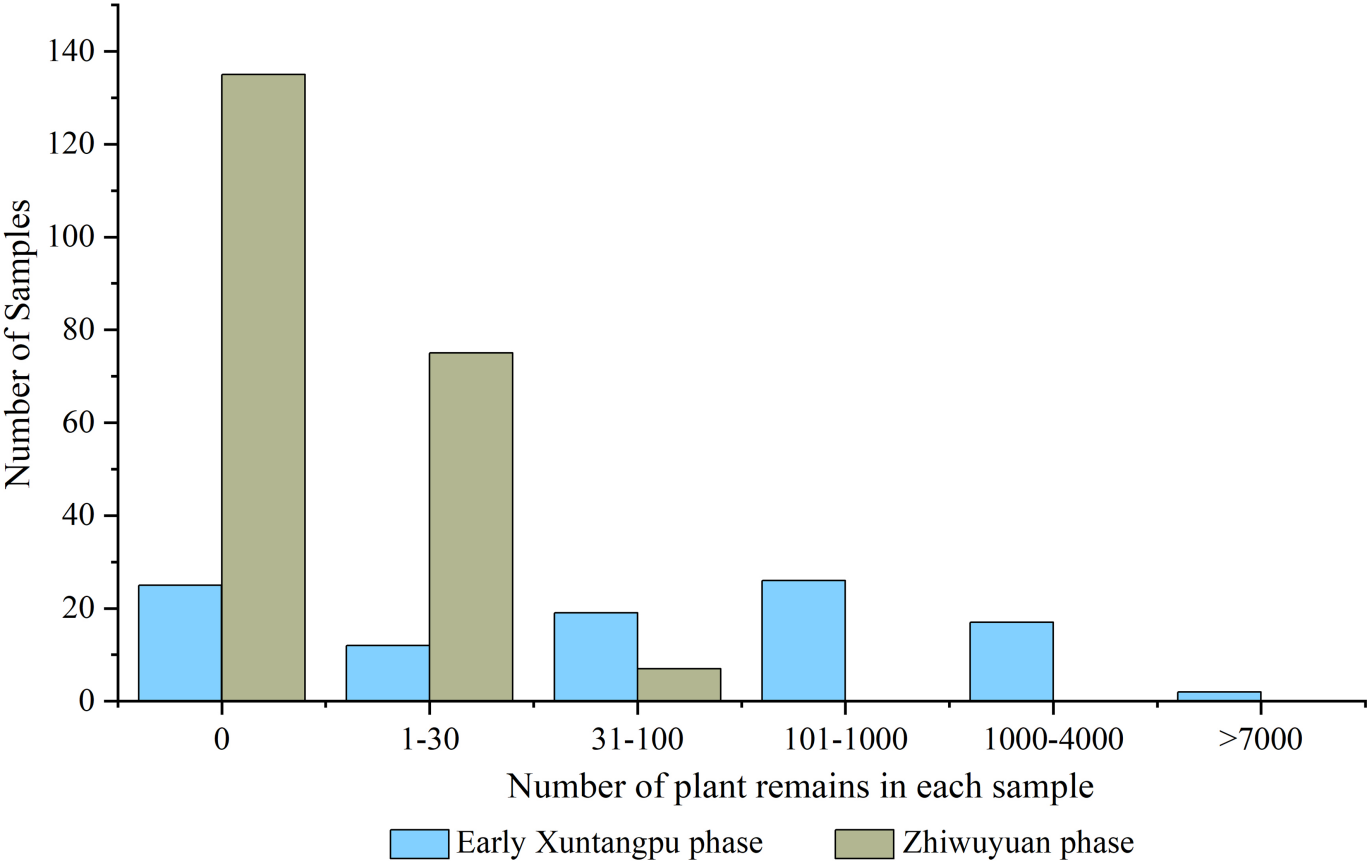
Abundance distribution of plant remains from the Zhiwuyuan site.

The preservation conditions of plant remains from Zhiwuyuan varied greatly between samples and periods. In total, 67,918 plant remains have been recovered in this study, including 67,165 from the Early Xuntangpu period and 753 from the Zhiwuyuan period (Supplementary Table S1). Moreover, 25 of 101 (24.7%) collected samples from the Early Xuntangpu phase and 135 of 217 (62.2%) from the Zhiwuyuan phase yielded no plant remains at all, and significant internal differences likewise could be observed in the abundance of plant remains in other samples (Figure 4).

**Figure 5:**
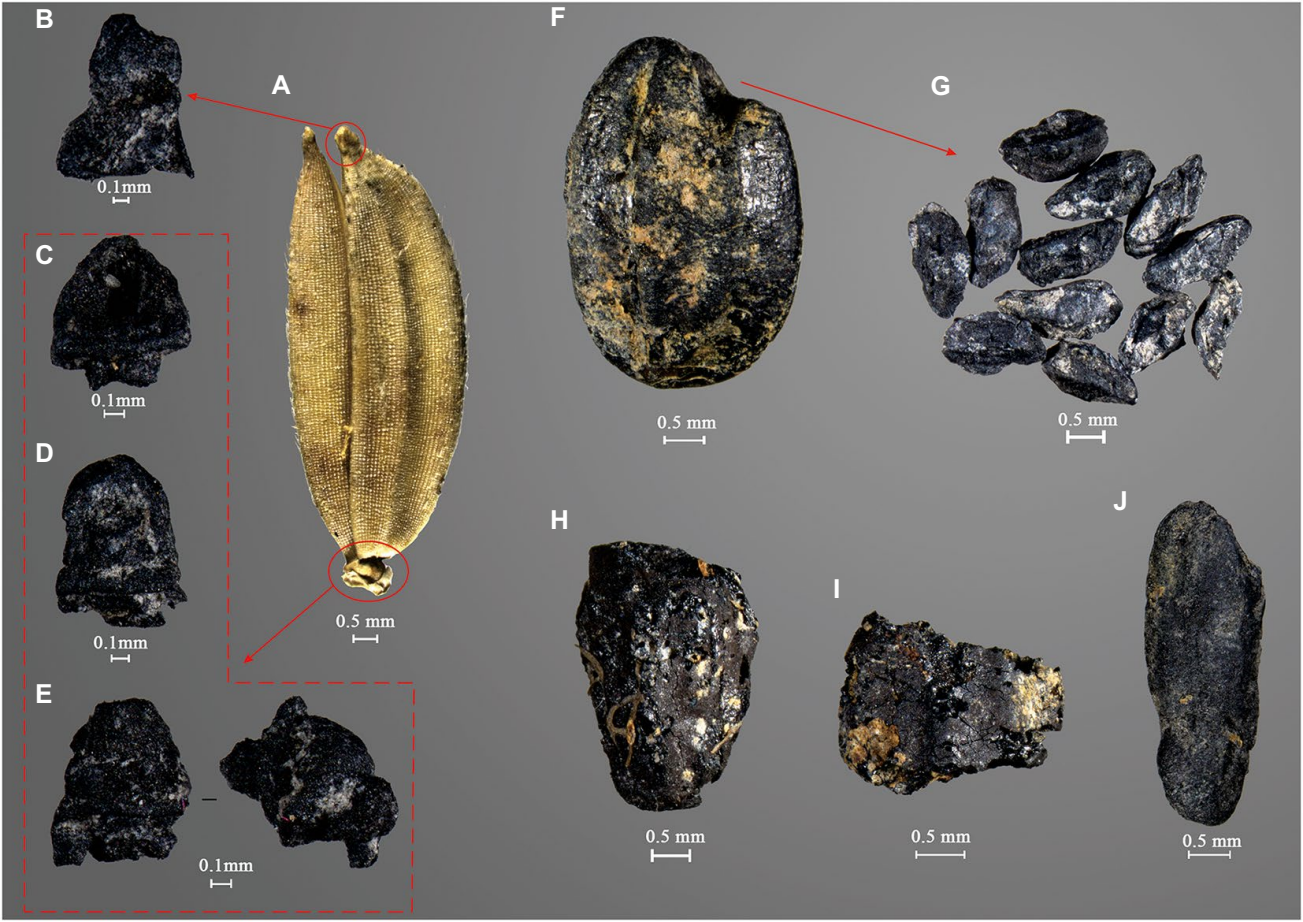
Different types of rice remains from the Zhiwuyuan site. **(A)** Modern rice with husk, **(B)** apex of rice husk, **(C)** rice spikelet base (non-shattering type), **(D)** rice spikelet base (shattering type), **(E)** rice spikelet base (protruding type), **(F)** mature rice grain, **(G)** embryos of rice grain, **(H)** big fragment of rice grain, **(I)** small fragment of rice grain, and **(J)** immature rice grain.

Overall, 38 taxa of plants have been identified to species, genus, or family levels. All these remains could be grouped into four categories: crops, fruits, grasses, and other weeds. Rice (*Oryza sativa*) and foxtail millet (*Setaria italica*) are the two crops recovered from Zhiwuyuan. The noteworthy point is that quite diversified rice remains have been identified, comprising grains, spikelet bases, apexes of husk, and isolated embryos (Figure 5). Rice grains could be classified into intact grains, large fragments (nearly half or larger), and small fragments (smaller than half) according to their preservation conditions, while the intact grains included mature and immature types. In total, 59,190 of these remains have been identified in this study, accounting for 87.15% of all plant remains. Rice spikelet base is the most abundant type, of which 44,914 specimens have been recovered. In addition, 22 intact mature rice grains have been measured individually, producing averages of length at 4.03 mm, width at 2.50 mm, and thickness at 1.83 mm. These rice grains from Zhiwuyuan, Dalongdong, and Anhe showed an explicit feature of short-grained type (for details, see the section “Discussion” below and Supplementary Table S3).

**Figure 6:**
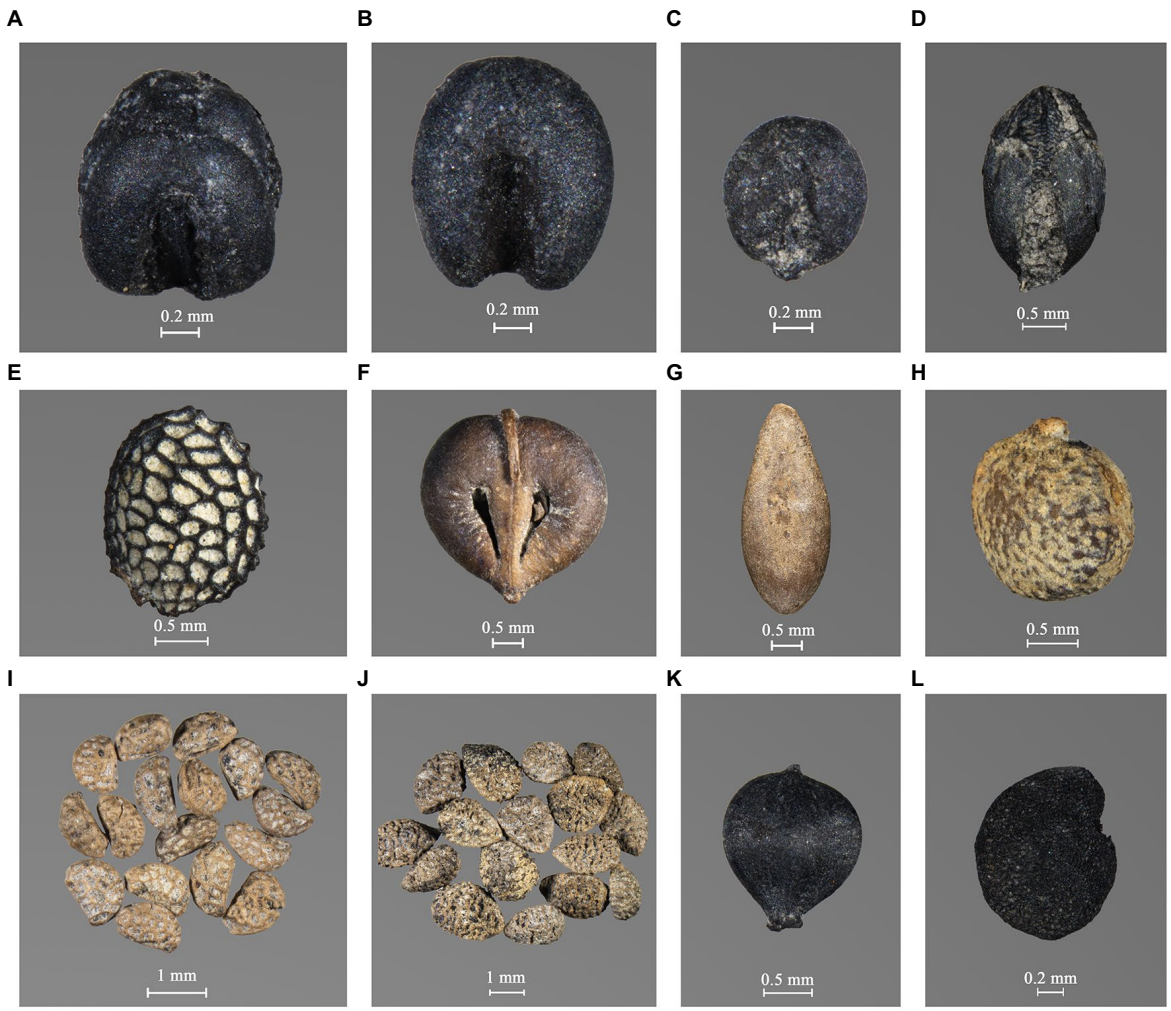
Other main plant remains from the Zhiwuyuan site. **(A)** Mature foxtail millet grain, **(B)** immature foxtail millet grain, **(C)** very immature foxtail millet grain, **(D)** *Setaria* sp., **(E)** *Actinidia* sp., **(F)** *Vitis* sp., **(G)** *Cucumis* sp., **(H)** *Broussonetia papyrifera*, **(I)** *Rubus* sp., **(J)** *Sambucus* sp., **(K)** *Scirpus* sp., and **(L)** Solanaceae.

Similarly, foxtail millet grains could be classified into mature, immature, and very immature categories (Figures 6A–C), according to previous research and criteria (Song et al., 2013; Deng et al., 2021). Apart from these intact grains, fragments of mature and immature grains were identified. Only 1,626 foxtail millet grains and fragments have been recovered, accounting for 2.39% of all plant remains.

Fruit remains from the Zhiwuyuan site are rich, referring to 6.41% of all plant remains. However, 3,569 are *Sambucus* sp. (Figure 6J), taking up 81.99% of this category. Besides, *Rubus* sp. (Figure 6I) and *Broussonetia papyrifera* (Figure 6H) are also relatively common in the Zhiwuyuan samples, especially during the Early Xuntangpu phase, while other fruits like *Diospyros* sp., *Actinidia* sp. (Figure 6E), *Vitis* sp. (Figure 6F), and *Cucumis* sp. (Figure 6G) appeared only occasionally and in low quantities.

Grasses are dominated by *Setaria* sp. (Figure 6D) and *Digitaria* sp., with the sparse discovery of *Echinochloa* sp., *Eleusine indica*, and other Panicoideae or Pooideae seeds. The total number of these remains is only 250, accounting for 0.37% of all plant remains. Regarding other weeds, 1,088 Caryophyllaceae seeds have been discovered, but most are from three collected samples. *Scirpus* sp. (Figure 6K) is the most common weed from the Zhiwuyuan site, while Brassicaceae, Solanaceae (Figure 6L), Polygonaceae, and other weeds appeared in limited quantities. Overall, grasses and other weeds are not abundant in most samples in Zhiwuyuan, accounting for 4.05% of all plant remains in total. The current evidence allowed only limited insights into the nature of crop cultivation, like dryland, rainfed, and irrigated permanent fields.

## Discussion

### Farming Practices of the Neolithic Zhiwuyuan in Northern Taiwan

**Figure 7:**
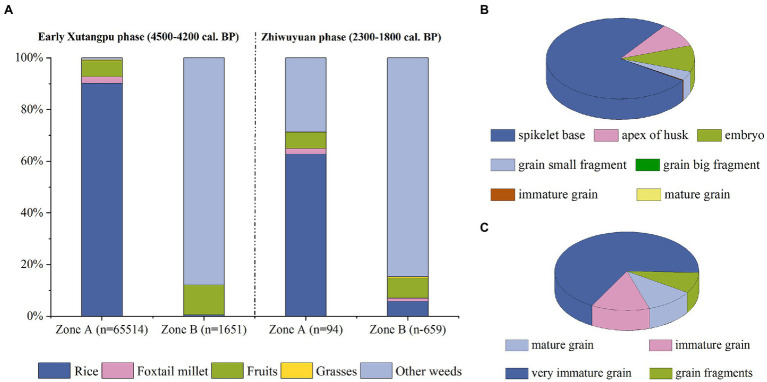
Assemblage of plant remains from the Zhiwuyuan site. **(A)** Proportions of different plant remains of the Early Xuntangpu phase and Zhiwuyuan phase from Zone A and B, **(B)** proportions of different types of rice remains of the Early Xuntangpu phase from Zone A, and **(C)** proportions of different kinds of foxtail millet remains of the Early Xuntangpu phase from Zone A.

The Zhiwuyuan site provides a precious chance to investigate early farming in Taiwan. In the Early Xuntangpu archaeological context, many plant remains have been recovered, but obvious spatial differentiation could be observed across the site. As shown in Figure 7A, assemblages of plant remains in the two sampled zones showed significant differences, wherein 97.54% of plant remains are from Zone A. This high concentration in one versus another zone could reflect possible different working areas that serviced different functions in the ancient settlement. The assemblages of plant remains further could clarify this scenario, noting that Zone A included mostly crops, while Zone B was dominated by varied weeds, with only three small fragments of rice grains and seven spikelet bases (Figure 7A).

Moreover, most rice remains from Zone A are spikelet bases, apexes of husks, and isolated embryos (Figure 7B), while only 46 mature rice grains have been recovered. A similar pattern could be observed with foxtail millet, of which immature and very immature grains accounted for 80.63% of the whole assemblage (Figure 7C). Overall, the high proportion of byproducts suggests that this area was used for threshing, dehusking, and winnowing cereal crops.

The proportions of rice and millet are difficult to speculate in the contemporary farming practice based solely on a direct comparison of absolute quantities, because the remains of spikelet bases, isolated embryos, and husk fragments of foxtail millet usually are too small to be preserved in archaeological records. As a result, the abundant discovery of the rice remains cannot lead to a simple conclusion of a rice-dominated crop pattern. Notably, the number of mature foxtail millet grains is much higher than rice. Even when considering immature and very immature grains, the total amount of foxtail millet still is higher than the intact grains of mature and immature rice. Hence, foxtail millet quite possibly played an essential role in the Neolithic subsistence of northern Taiwan.

Since its early phase, the main subsistence strategy at Zhiwuyuan has been cereal crop cultivation. All of the fruit remains from this site are from fleshy types, and no starchy nuts such as acorns were present, noting that starchy nuts otherwise are quite common in hunting-gathering sites (e.g., Rosenberg, 2008; Tushingham and Bettinger, 2013). Moreover, the most abundant fruit, *Sambucus* sp., was possibly a natural shrub in the settlement and not necessarily a food resource. A similar crop pattern continued into the Zhiwuyuan phase. However, the quantity of plant remains in this period decreased dramatically compared to the previous Early Xuntangpu phase. The total amount of all types of rice remains is only 97, and three mature grains and seven immature grains of foxtail millet have been recovered. These findings may reflect the effects of a change in settlement planning or poor preservation conditions.

Overall, archaeobotanical evidence from the Zhiwuyuan site indicates a dating of no later than 4,500 cal. BP for the cultivation of rice and foxtail millet as staple foods in northern Taiwan, and this mixed farming strategy continued at least as late as 1,800 cal. BP at this site.

### Rice Variety Cultivated in Neolithic Taiwan as Revealed by Morphometrics

**Figure 8:**
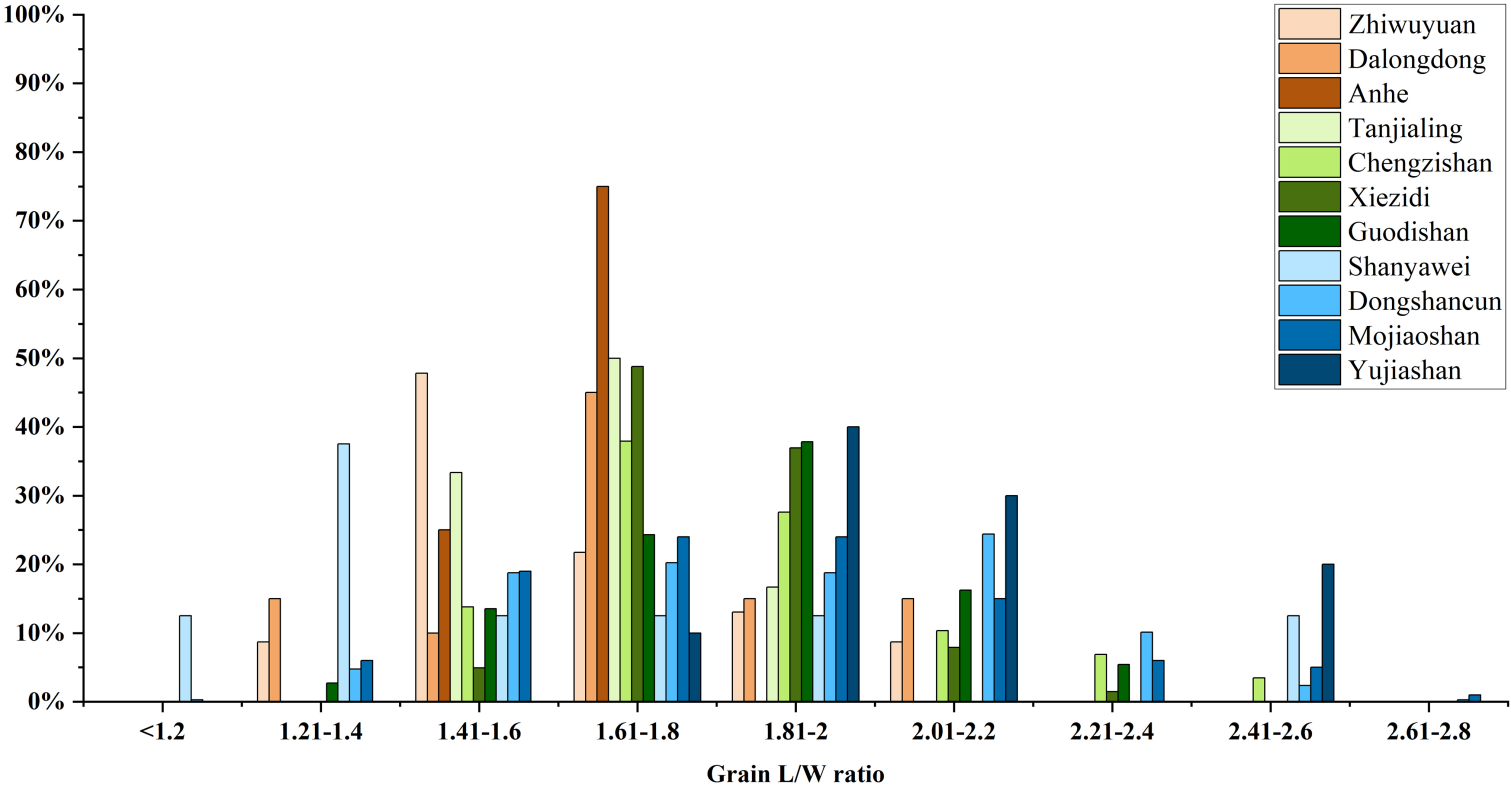
Rice grain L/W ratio distributions of Zhiwuyuan, Dalongdong, and Anhe compared to remains from Neolithic sites of middle and lower Yangtze valley.

Previous research has stressed that morphometric analysis of rice grains cannot be used for discrimination of domesticated vs. wild rice due to complex factors, but it still could provide practical indications to distinguish *japonica* vs. *indica* varieties under the premise of domestic species in archaeobotanical research (Fuller et al., 2007; Castillo, 2011). Generally, the length-width (L/W) ratios of >2.2 indicate *indica*-type rice, and ratios <2.0 are *japonica* type, although such tendencies are complex and by no means absolute. This criterion has been attested by the integrated study of ancient DNA and grain morphometrics in archaeological sites of Thailand and India (Castillo et al., 2016). According to this criterion, rice grains from Zhiwuyuan, Dalongdong, and Anhe showed typical features of *japonica* rice, with L/W ratios of most specimens under 2.0 and none at all above 2.2 (Figure 8). Comparison with other contemporary discoveries from the middle and lower Yangtze valley reveals a similar pattern, supporting the hypothesis that early rice cultivated in East Asia and Southeast Asia were all of the *japonica* type (Fuller, 2011).

**Figure 9:**
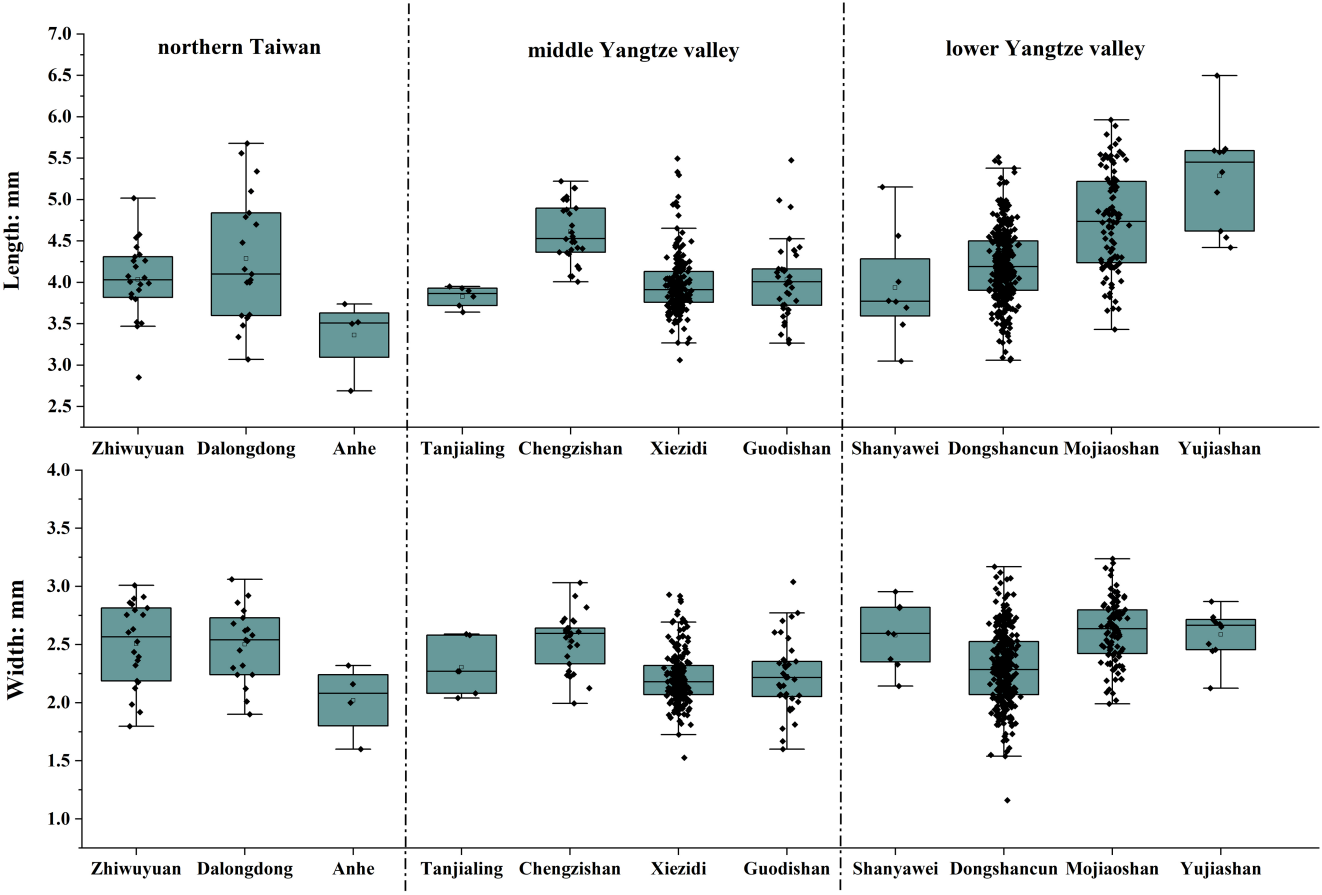
Comparison of length and width of rice grains from Zhiwuyuan, Dalongdong, Anhe, and other late Neolithic sites (*ca.* 4,800–4,200 cal. BP) in the middle and lower Yangtze valley.

A more detailed analysis suggests that the rice variety cultivated in Neolithic northern Taiwan was a typical short-grained type. The average lengths of rice grains from Zhiwuyuan and Dalongdong are 4.03 and 4.29 mm, respectively (Figure 9). This value is similar to contemporary remains from the middle Yangtze valley. For example, the average lengths of rice grains from the Tanjialing locality of Shijiahe city ruins (Deng et al., 2013), Xiezidi (Tang et al., 2014), and Guodishan (Deng et al., 2020b) usually are shorter than 4.5 mm (Deng, 2015). However, most of the lower Yangtze valley remains are much longer, falling in the range of 4.5–6 mm (Gao, 2017), although some short-grained types are found in the northern part of the Yangtze plain (for example, some sites like Dongshancun in Jiangsu province), and mountainous regions in the south that are culturally connected to inland Jiangxi and Fujian provinces (e.g., Shanyawei; Figure 9). On the other hand, no noticeable difference could be observed in the widths of rice grains from different regions (Figure 9), which indicates the northern Taiwan and middle Yangtze valley varieties were short-grained but plump types. In this case, the four rice grains from the Anhe site were possibly immature, as their lengths and widths were significantly lower than others.

### The Beginning of Agriculture in Taiwan

As stated above, the emergence of agriculture in Taiwan is of great significance for investigating the ancient Austronesian population expansion. However, owing to the lack of direct evidence, many previous discussions on this issue were based on the discoveries at the single site of Nanguanlidong (e.g., Tsang, 2005; Hsieh et al., 2011; Tsang et al., 2017), generally regarded as the earliest farming site in Taiwan. Unfortunately, the rice and millet remains from Nanguanlidong were not dated directly, and the precise context information often was unspecified in terms of association with a particular archaeological layer and locality. Nine radiocarbon dates from Nanguanlidong have been published, but eight of those results were conventional charcoal samples that produced wide error ranges of 300–800 years (Figure 3D). Moreover, radiocarbon dates of different phases at Nanguanlidong do not fit the cultural sequence, for example, noting that most dates from distinctly different contexts of Layer 3 and Layer 1 fall into the same time range. As a result, the available radiocarbon dates from Nanguanlidong have not pinpointed the exact time of the first farming in Taiwan.

As for Zhiwuyuan and Dalongdong, the direct dates of crop remains are generally consistent with other charcoal samples, although some conventional dates show a wide range. Generally, the Bayesian model analysis suggests a starting date around 4,600 cal. BP at both sites (Figures 3A,B).

Eight AMS radiocarbon dates have been obtained from the Anhe site, including two from human bones and six from charcoal (Figure 3C; Supplementary Table S2). According to the stratigraphy information of these dated contexts, the two old dates from contexts WTP2L18 and WTP1L12 should be excluded as they contradicted their stratigraphic order and therefore may have been old or displaced materials. Hence, the reliable starting date of human activities and farming practice at the Anhe site was around 4,800–4,600 cal. BP.

The emergence of farming in Taiwan was perhaps not as early as previously has been suggested at 5,000 cal. BP or older. In fact, our new proposed date of earliest farming at 4,800–4,600 cal. BP in Taiwan accords well with the earliest such discoveries from the adjacent regions. The full cross-regional sequence can be outlined with farming in Fujian and Guangdong around 5,000–4,800 cal. BP, followed by evidence in Taiwan about 4,800–4,600 cal. BP, next dispersing southward to Northern Luzon at 4,200–4,000 cal. BP, and later evident in Sulawesi around 3,500 cal. BP (Deng et al., 2018b, 2020a, 2022; Yang et al., 2018; Hung et al., 2022).

### The Important Role of Millets and the Mixed Farming

Regarding the specific farming strategies practiced by early Austronesians in Taiwan, the prior research at Nanguanlidong already has emphasized the importance of mixed farming instead of pure rice cultivation (Tsang et al., 2017). Now, the crop remains from Zhiwuyuan further have confirmed this pattern, evident not only in southwestern Taiwan but also in the northern part. Nevertheless, a clear difference still could be found between the two sites, as no broomcorn millet has been recovered from Zhiwuyuan. Therefore, more basic work from other sites in the future will be important to clarify the site-specific variations within the overall pattern.

Stable isotope data of human bones from 11 sites in Taiwan demonstrated that millets were an important staple food in the Neolithic period (4,600–2,000 cal. BP) and Iron Age (2,000–400 cal. BP; Lee et al., 2018). For example, the δ^13^C values of six individuals from the Yuanshan site (*ca.* 3,200–2,300 cal. BP) are generally higher than −16%, and the δ^14^N are under 10‰, suggesting a high proportion of C_4_ millets in their daily diet. In addition, millets were used as food for domesticated animals such as pigs and dogs at this site (Lee et al., 2016).

**Figure 10:**
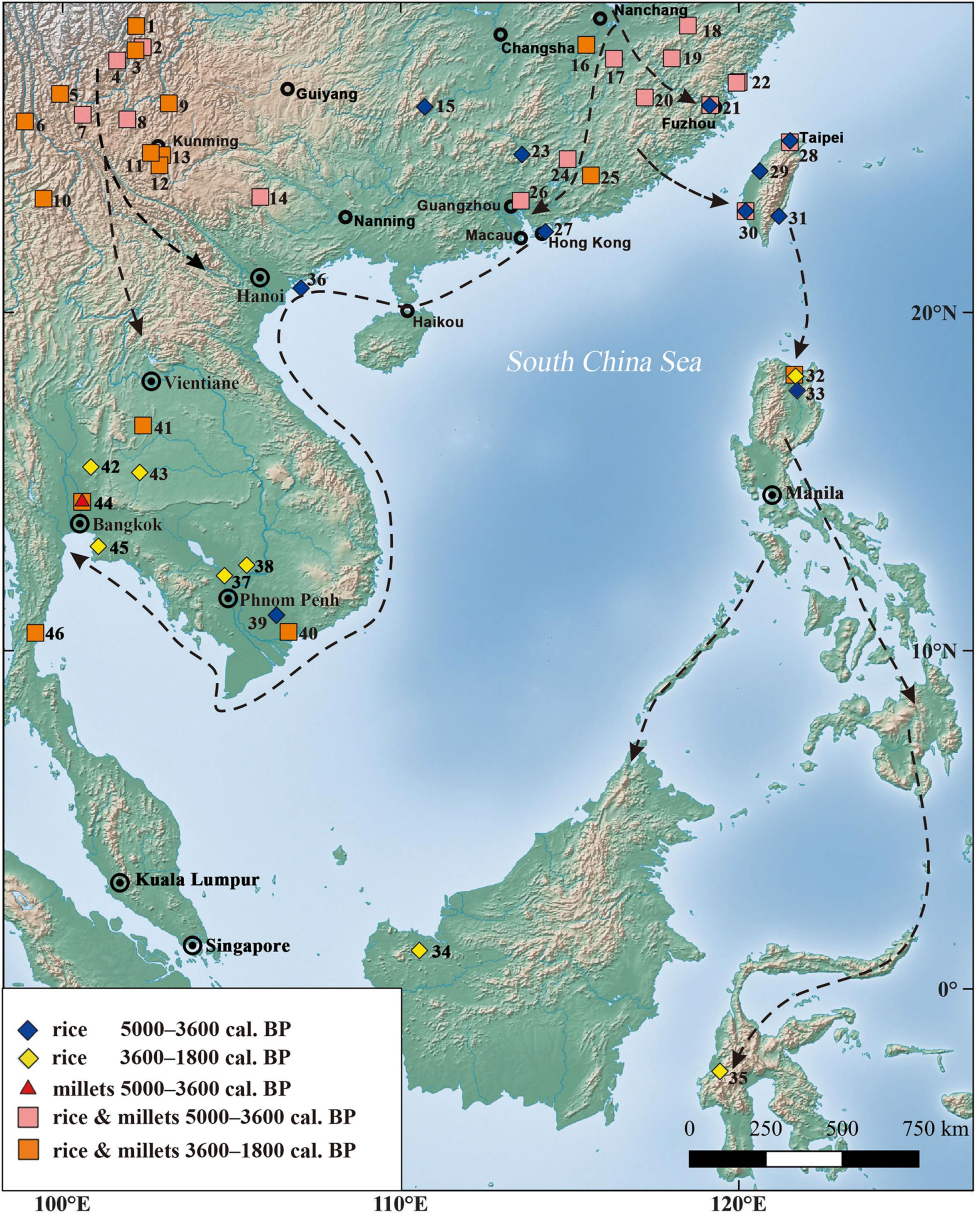
Distribution of major sites with rice and/or millets in Southern China and Southeast Asia, and related agriculture dispersal routes; (1) Gaopo, (2) Henglanshan, (3) Shapingzhan, (4) Guijiabao, (5) Haimenkou, (6) Shilinggang, (7) Baiyangcun, (8) Dadunzi, (9) Yubeidi, (10) Shifodong, (11) Heposuo, (12) Guangfentou, (13) Xueshan, (14) Gantuoyan, (15) Xiaojin, (16) Niucheng, (17) Guodishan, (18) Shanyawei, (19) Hulushan, (20) Nanshan, (21) Zhuangbianshan and Baitoushan, (22) Pingfengshan and Huangguashan, (23) Shixia, (24) Laoyuan, (25) Shixiongshan, (26) Gancaoling, (27) Shaha, (28) Zhiwuyuan (rice and foxtail millet) and Dalongdong (only rice), (29) Anhe, (30) Nanguanlidong and Youxianfang (rice and millets) and Nanguanli and Sanbaozhunan (only rice), (31) Chaolaiqiao, (32) Nagsabaran and Magapit, (33) Andarayan, (34) Gua Sireh, (35) Minanga Sipakko, (36) Cai Beo, (37) Samrong Sen, (38) Krek 52/62, (39) An Son, Loc Giang, (40) Rach Nui, (41) Non Nok Tha, (42) Khok Charoen, (43) Ban Non Wat, (44) Non Pa Wai, Non Mak La, and Nil Kham Haeng, (45) Khok Phanom Di and Nong Nor, and (46) Khao Sam Kaeo (Details of all sites and references are listed in Supplementary Table S4).

The corroborating lines of evidence demonstrate the importance of millets in the Neolithic and Iron Age agricultural systems of Taiwan. These findings are consistent with recent discoveries in the southeast and southern coast of mainland China, where both rice and millets were cultivated after 5,000 cal. BP, for example as reported at Nanshan in Fujian (*ca.* 5,000 cal. BP; Yang et al., 2018) and at Gancaoling in Guangdong (4,800 cal. BP; Deng et al., 2022). In northern Luzon, foxtail millet has been reported from the Metal Age layer of Nagsabaran, although the earliest appearance in this region remains uncertain (Hung et al., 2022; Figure 10). One probable millet (Setaria) seed, *ca.* 3200–3800 cal. BP, was found at Uai Bobo 2 in East Timor (Glover, 1986).

Similar conditions could be found in Mainland Southeast Asia. Residue analysis of stone tools from the Cai Beo site in the Ha Long Bay of coastal northeastern Vietnam revealed the arrival of rice in this region during the period of 4,500–4,000 cal. BP (Wang et al., 2022), and millet likely came simultaneously with rice. On the southern coast of Vietnam, rice spikelet bases dated to 4,100–3,200 cal. BP have been confirmed by micro-CT analysis of pottery sherds from Loc Giang and An Son (Barron et al., 2017). More importantly, direct dating of foxtail millet from the Non Pa Wai site indicated these crops had arrived on the south coast and nearby inland area of Thailand around 4,400–4,100 cal. BP (Weber et al., 2010). New evidence demonstrated that rice and millets likely dispersed into the coastal regions of Vietnam and Thailand around the same time (Figure 10, references in Supplementary Table S4), although more targeted work still will be needed to clarify details of the process of farming expansion.

Overall, archaeobotanical studies in recent years have suggested that rice and millets were staple crops for Neolithic populations in southern China, Taiwan, and Southeast Asia. This strategy has continued for thousands of years, as previously mentioned in several ethnographical records from the early twentieth century of Taiwan and Southeast Asia (Kano, 1946).

## Conclusion

The dietary subsistence of Neolithic Taiwan is at the central place of debates about the primary motivation of ancient Austronesian migrations. This present study provides the first directly dated and the earliest evidence of Neolithic farming activities from northern Taiwan. The direct AMS radiocarbon dates of the crop remains in the early period now confidently can confirm to be at least 4,500 cal. BP for the cultivation of rice and foxtail millet at Zhiwuyuan in northern Taiwan. However, a more comprehensive examination of previous radiocarbon dates from related sites suggests that the emergence of agriculture on the island was most probably around 4,800–4,600 cal. BP.

The new findings significantly verify that rice and millet were cultivated together by early Austronesians in most parts of Taiwan ever since its early Neolithic phase. Within this overall pattern, localized differences in crop compositions and varieties still could be observed at specific sites and regions.

Moreover, the morphometric analysis suggested that rice cultivated in Zhiwuyuan, Dalongdong, and Anhe was a short-grained but plump type of *japonica* rice, similar to contemporaneous discoveries from the middle Yangtze valley and the mountainous region of southeast China. Our results from both the morphometric study and radiocarbon dates all corroborate the long-term chronological sequence and larger cross-regional picture of the ancient dispersals of farmers and their farming traditions and cultures, from the middle Yangtze valley *via* the southeast coast of mainland China, next to Taiwan, and then Island Southeast Asia.

## Data Availability Statement

The original contributions presented in the study are included in the article/Supplementary Material, further inquiries can be directed to the corresponding authors.

## Author Contributions

ZD and HCH designed the study. SCK conducted archaeological excavation and sample collection. ZD completed sample processing and identification and analyzed the data. ZD, HCH, and MC wrote the manuscript. All authors contributed to the article and approved the submitted version.

## Funding

This work was supported by the National Natural Science Foundation of China (Grant Nos. T2192953 and 41872027), the Strategic Priority Research Program of Chinese Academy of Sciences (Grant No. XDB26000000), the Australian Research Council (Grants DP1501044 and DP190101839), and the Chiang Ching-kuo Foundation for International Scholarly Exchange (Grant RG021-P-10).

## Conflict of Interest

The authors declare that the research was conducted in the absence of any commercial or financial relationships that could be construed as a potential conflict of interest.

## Publisher’s Note

All claims expressed in this article are solely those of the authors and do not necessarily represent those of their affiliated organizations, or those of the publisher, the editors and the reviewers. Any product that may be evaluated in this article, or claim that may be made by its manufacturer, is not guaranteed or endorsed by the publisher.
